# Genetic Analysis of Norovirus GII.4 Variant Strains Detected in Outbreaks of Gastroenteritis in Yokohama, Japan, from the 2006-2007 to the 2013-2014 Seasons

**DOI:** 10.1371/journal.pone.0142568

**Published:** 2015-11-06

**Authors:** Makoto Kumazaki, Shuzo Usuku

**Affiliations:** Microbiological Testing and Research Division, Yokohama City Institute of Public Health, Kanagawa, Japan; The University of Hong Kong, HONG KONG

## Abstract

Noroviruses (NoVs) are the leading cause of acute gastroenteritis, both in sporadic cases and outbreaks. Since the 1990s, the emergence of several GII.4 variants has been reported worldwide. To investigate the epidemic status of NoV, 6,724 stool samples collected from outbreaks in Yokohama, Japan, from the 2006–2007 to 2013–2014 seasons were assessed for NoVs. We genotyped one specimen from each GII outbreak and conducted a sequence analysis of the VP1 gene for several GII.4 strains. Of the 947 NoV outbreaks during our study, GII was detected in 835, and GII.4 was the predominant genotype of GII. Five different GII.4 variants, Yerseke 2006a, Den Haag 2006b (2006b), Apeldoorn 2007, New Orleans 2009, and Sydney 2012, were detected. During this study period, the most prevalent variant of GII.4 was 2006b, and in each individual season, either 2006b or Sydney 2012 was the predominant variant. Out of the 16 detected 2006b strains, 12 had some amino acid substitutions in their blockade epitope, and these substitutions were concentrated in three residues. Two of the 2006b strains detected in the 2012–2013 season had a S368E substitution, which is consistent with the amino acid residues at same site of NSW0514 (Sydney 2012 prototype). Among the 16 detected strains of Sydney 2012, a phylogenetic analysis showed that all five strains detected in Yokohama during the 2011–2012 season clustered away from the other Sydney 2012 strains that were detected in the 2012–2013 and 2013–2014 seasons. These five strains and other Sydney 2012 strains in Yokohama had a few amino acid differences in the blockade epitopes compared with NSW0514. The amino acid substitutions observed in this study provide informative data about the evolution of a novel GII.4 variant.

## Introduction

Noroviruses (NoVs) are the most frequent cause of acute gastroenteritis worldwide among people of all ages [1, 2]. They are single-stranded positive-sense RNA viruses in the family Caliciviridae. The NoV genome is 7.5 kb long and encodes three open reading frames (ORFs), a nonstructural protein (ORF1) and two structural proteins (ORF2, which encodes VP1, and ORF3, which encodes VP2). Based on their VP1 gene, NoV strains can be classified into six genogroups (G), GI–GVI, of which GI, GII, and GIV infect humans [1]. NoV GI contains nine genotypes, and NoV GII contains 22 genotypes [3].

Globally, NoV GII strains are dominant, and GII.4 has been the predominant NoV genotype. Genetically distinct novel GII.4 variants have emerged every two to three years and spread rapidly around the world [4–7]. GII.4 variants US95/96, Farmington Hills 2002, Hunter 2004, Den Haag 2006b (2006b), New Orleans 2009, and Sydney 2012 are recognized as pandemic variants, while some variants, such as Asia 2003 and Yerseke 2006a, have been reported only in limited regional epidemics [3, 4, 6]. Moreover, it has been reported that GII.4 causes a more severe gastroenteritis than other genotypes [8, 9]. To determine the reason for the predominance of GII.4 and its increased disease severity, an analysis of the viral antigenicity and pathogenicity of GII.4 is needed. Unfortunately, an efficient culture system for human NoVs has not yet been developed.

Structural analyses show that VP1 can be divided into two distinct domains, the shell (S) domain and the protrusion (P) domain of the capsid. The P domain can be further divided into the P1 and P2 subdomains [10]. P2 is a hypervariable domain that contains the putative receptor-binding sites [11, 12]. Using bioinformatic approaches, five antibody epitopes (epitope A–E) on the surface of the GII.4 P2 subdomain were predicted, and the emergence of pandemic strains is often associated with alterations in these epitopes [13, 14].

To our knowledge, longitudinal studies of GII.4 strains derived from outbreaks in Japan have not been reported, although similar studies have been reported for other countries [5, 6, 15–18]. In this study, to determine the trends of circulating NoV strains and to investigate the characteristics of GII.4 variant strains, we performed a genetic analysis of the strains detected in NoV outbreaks in Yokohama. This area has a population of about 3,700,000, and it is located in the center of Japan on the coastline of the Pacific Ocean. We believe this is the first study to conduct long-term monitoring of GII.4 variants in Yokohama, Japan.

## Materials and Methods

### Ethics Statement

All procedures in this study that involved human participants were performed in accordance with the ethical standards of the institutional research committee of Yokohama City Institute of Public Health, Kanagawa, Japan, and with the 1964 Helsinki declaration and its later amendments or with comparable ethical standards. Ethical clearance by the institutional research committee of Yokohama City Institute of Public Health is not needed because this study was conducted as outbreaks investigation for identifying causative agent of gastroenteritis. The patient information was anonymized and de-identified prior to analysis.

### Sample collection

Outbreaks of gastroenteritis in Japan are reported to local government public health centers by order of the Ministry of Health, Labour and Welfare. The local government public health centers then conduct field investigations. Between September 2006 and August 2014, 947 outbreaks of NoV gastroenteritis, suspected to be due to foodborne or person-to-person transmission, were reported, and 6,724 stool samples were collected for NoV testing by the Health and Social Welfare Bureau, Yokohama, Japan. These samples were mainly collected in primary schools, nursing homes for the aged, kindergartens/nursery schools, hospitals, welfare facilities, and restaurants. A NoV outbreak was defined as two or more cases of acute gastroenteritis occurring in a given setting, and a NoV season was defined as the 12-month period from September through August of each year.

### Detection of NoV gene

A 10% stool suspension was prepared by mixing each stool sample with phosphate-buffered saline, followed by centrifugation at 10,000 × g for 10 min at 4°C. Viral RNA was extracted from the supernatants with the RNeasy Mini Kit (Qiagen, Hilden, Germany). Real-time RT-PCR detection of NoV was performed with a Smart-Cycler II (Cepheid, Sunnyvale, CA, USA) using a QuantiTect Probe RT-PCR Kit (Qiagen) with separate reactions for NoV genogroups I and II. The primers and probes used to detect these viruses have been described in other reports [19, 20].

### RT-PCR for NoV GII genotyping and NoV GII.4 variant typing

One positive specimen selected randomly from each NoV GII outbreak was subjected to gene amplification of region C (the 5' end of the ORF2 gene) to determine the GII genotype and the GII.4 variant type using a web-based Norovirus Genotyping Tool Version 1.0 [21]. RT-PCR was performed with a TaKaRa One Step RNA PCR Kit (Takara Bio Inc., Shiga, Japan). The primers used for PCR have been described in other reports [22, 23].

### PCR for the VP1 region of GII.4 strains

For the representative strains of GII.4 variant types, 2006b and Sydney 2012, we conducted a further sequence analysis of the VP1 gene. The cDNA was synthesized from the extracted viral RNA with SuperScript III Reverse Transcriptase (Invitrogen, Carlsbad, CA, USA) and random hexamer primers (Takara Bio Inc., Shiga, Japan), for use as the template for PCR. Then, PCR was performed to amplify the VP1 genes of NoV GII.4 with TaKaRa Ex Taq DNA polymerase (Takara Bio Inc., Shiga, Japan). We used the COG2F and TX30SXN primers for the first PCR and COG2F, R5841 (5ʹ-GACCCGTGAACAACTTTTCCAAAG-3ʹ), LVPF, LVCAPEND, F6558 (5ʹ-CATAAATCAGGCTATGTYACAGTRGC-3ʹ), F6565 (5ʹ- CAGGCTATGTCACAGTGGCTCACA-3ʹ), and TX30SXN primers for the second PCR [16, 24].

### Sequence and phylogenetic analysis

The nucleotide sequences of the purified PCR products (QIAquick PCR Purification Kit, QIAGEN) were determined using a BigDye Terminator Cycle Sequencing Kit (Applied Biosystems, Foster City, CA, USA) and a Genetic Analyzer 3130 (Applied Biosystems). The obtained data were used to construct a phylogenetic tree on the basis of amino acid sequences with the neighbor-joining method using MEGA 5 software (http://www.megasoftware.net/) with 1,000 bootstrap replicates. The sequences reported in this paper have been deposited in the DDBJ/GenBank/EMBL databases under accession numbers LC005704–LC005735.

## Results

### NoV outbreaks

Of the 947 NoV outbreaks during eight consecutive 12-month periods starting in September 2006, 835 (88.2%) were caused by NoV GII, 76 (8.0%) were caused by NoV GI, and 36 (3.8%) were caused by a mixture of NoV GI and GII. A summary of the NoV outbreaks in each season is listed in Table 1. The highest number of NoV outbreaks (155) occurred during the 2010–2011 season, and the lowest number (90) occurred during the 2009–2010 season. GII was dominant every season and consistently accounted for 79.8%–97.9% of the total NoV cases. Among the 835 outbreaks of GII, GII.4 was the most prevalent genotype, occurring in 483 outbreaks. GII.4 was the prevalent genotype in every season, except for the 2010–2011 season, in which GII.3 was the prevalent genotype. The GII.4 proportion of GII cases was more than 80% in the 2006–2007 and 2012–2013 seasons. In contrast, the GII.4 proportion of GII cases was less than 50% in the 2008–2009, 2009–2010, 2010–2011, and 2013–2014 seasons. Compared with its presence during other seasons, GII.6 was most prevalent in the 2008–2009 and 2013–2014 seasons. Similarly, GII.2 was most prevalent in the 2009–2010 and 2010–2011 seasons, and GII.14 was most prevalent in the 2011–2012 season.

**Table 1:** Number of norovirus outbreaks reported in Yokohama, Japan, from September 2006 to August 2014.

	No. of outbreaks								
Genogroup	Total (%)	Season							
Genotype		2006–2007	2007–2008	2008–2009	2009–2010	2010–2011	2011–2012	2012–2013	2013–2014
total	947 (100)	95	104	93	90	155	110	150	150
GI	76 (8.0)	2	13	14	14	8	6	10	9
Mixed GI and GII	36 (3.8)	0	8	1	4	7	7	6	3
GII	835 (88.2)	93	83	78	72	140	97	134	138
GII.2	77 (8.1)	3	9	1	24	27	3	6	4
GII.3	88 (9.3)	3	2	7	3	66	0	1	6
GII.4	483 (51.0)	75	61	36	36	35	65	116	59
GII.5	1 (0.1)	0	0	0	0	0	0	1	0
GII.6	96 (10.1)	0	0	30	1	2	3	2	58
GII.7	5 (0.5)	0	0	0	1	0	1	2	1
GII.12	11 (1.2)	0	0	1	3	4	2	0	1
GII.13	2 (0.2)	0	0	0	2	0	0	0	0
GII.14	53 (5.6)	3	9	3	2	2	22	4	8
GII.15	1 (0.1)	0	0	0	0	0	1	0	0
GII.17	2 (0.2)	0	0	0	0	0	0	1	1
GII ND^a^	16 (1.7)	9	2	0	0	4	0	1	0

^a^Not determined

### Detection of NoV GII.4 variants

**Fig 1:**
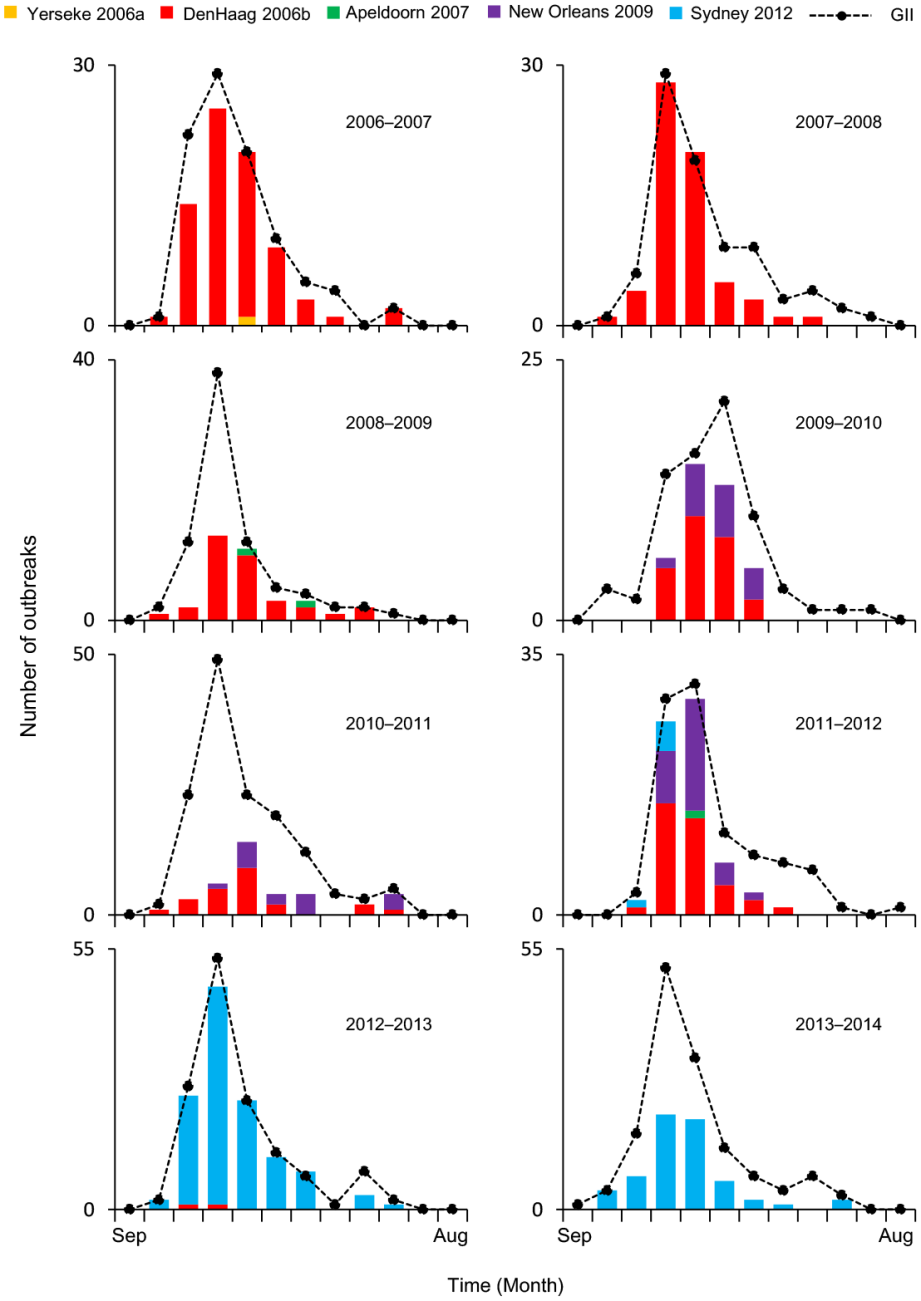
Monthly distribution of NoV GII.4 outbreaks by variant type in Yokohama, Japan. Monthly distribution of NoV GII.4 variants identified in NoV outbreaks in Yokohama, Japan, from the 2006–2007 season to the 2013–2014 season. The x axis shows the time course by month; the y axis shows the number of GII and GII.4 variant outbreaks. Yerseke 2006a (orange), Den Haag 2006b (red), Apeldoorn 2007 (green), New Orleans 2009 (violet), and Sydney 2012 (blue). The dashed line indicates transitions in the number of GII outbreaks.

Because GII.4 strains were the dominant type for most of the seasons in our study, we further analyzed their distribution and variant types. A total of 499 GII.4 strains that were detected in the GII.4 outbreaks (including 16 strains detected in mixed GI and GII outbreaks) were analyzed. The monthly distribution and variant types of the GII.4 strains from these outbreaks are shown in Fig 1. The GII.4 strains were mainly detected in winter and peaked in December–January annually. No GII.4 strains were detected in July, August, or September. Five different GII.4 variants were detected in Yokohama during this study period. The most prevalent variant was 2006b (257), followed by Sydney 2012 (183), New Orleans 2009 (55), Apeldoorn 2007 (3), and Yerseke 2006a (1). One or two of these variants were detected in each NoV season, except in the 2011–2012 season in which four different GII.4 variants were detected. In each season, either 2006b or Sydney 2012 was the predominant variant. 2006b was the predominant variant in the seasons between 2006 and 2012. In the 2012–2013 season, 2006b was identified for two outbreaks, and it has not been detected since January 2013. The first outbreak of Sydney 2012 occurred in November 2011, but Sydney 2012 was not the predominant variant in the 2011–2012 season, in which it was identified from only five outbreaks. Sydney 2012 spread rapidly from October 2012 and was the predominant variant in the 2012–2013 and 2013–2014 seasons. New Orleans 2009 was not the predominant variant in any of the NoV seasons, although it circulated in the seasons between 2009 and 2012. Yerseke 2006a and Apeldoorn 2007 variants were less common in all of the seasons during this study period in Yokohama.

### VP1 sequence and phylogenetic analysis of NoV GII.4 strains

**Fig 2:**
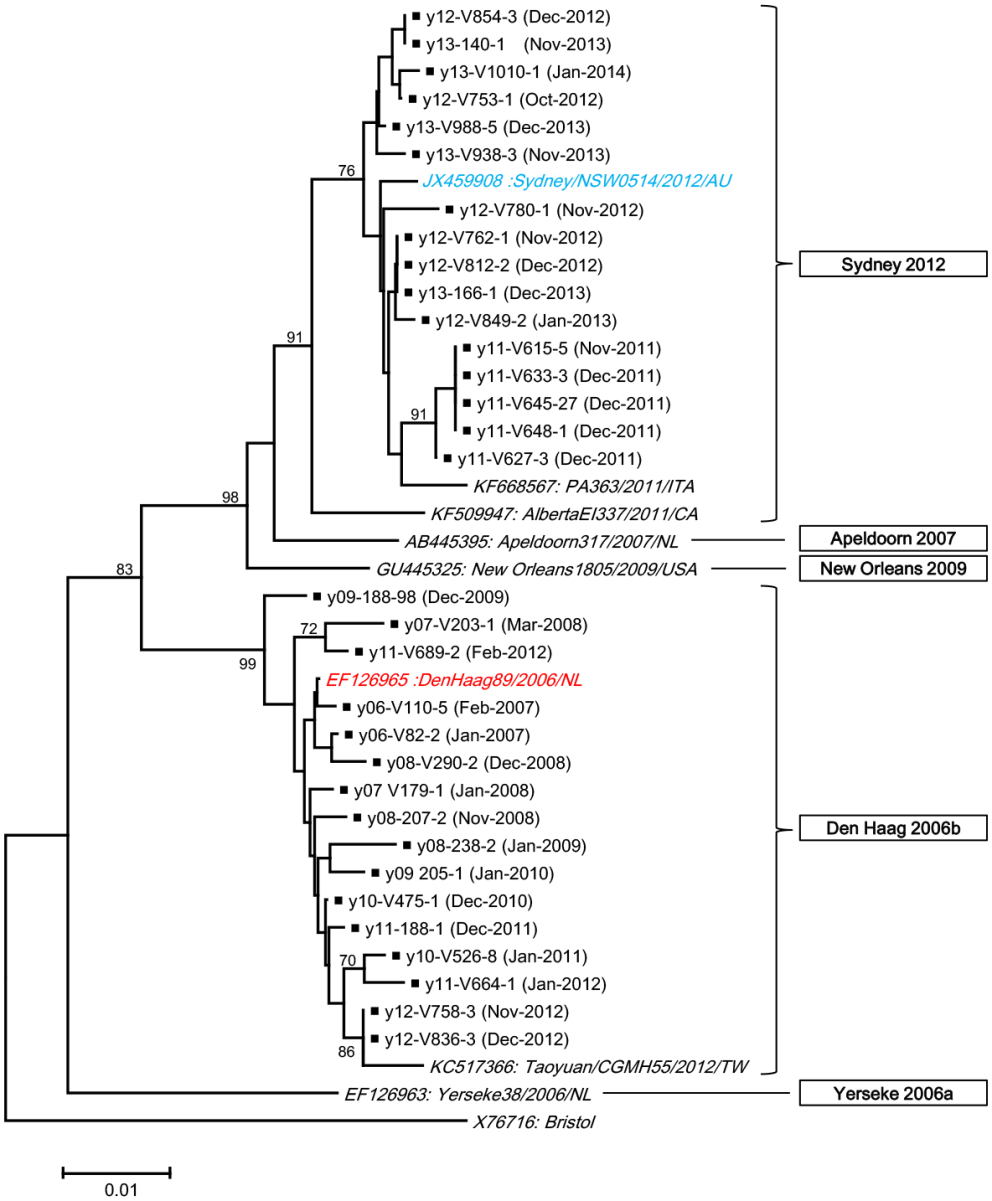
Phylogenetic tree based on the VP1 amino acid sequences of NoV GII.4 strains. A phylogenetic tree based on the VP1 amino acid sequences of NoV GII.4 strains. The tree was constructed with the neighbor-joining method using MEGA 5 software (http://www.megasoftware.net/) with 1,000 bootstrap replicates. The percentage of bootstrap support is indicated at each node (values < 70% are omitted). The scale bar represents the number of substitutions per site. Filled squares indicate strains of which the genes were analyzed in this study.

To clarify the genetic characteristics of the 2006b and Sydney 2012 strains which were predominant in Yokohama, we further analyzed these two variant type strains. The VP1 gene of representative strains of 2006b and Sydney 2012 variant types detected in Yokohama was analyzed by sequencing. The analyzed strains are listed in Table 2. The results of a phylogenetic analysis based on the amino acid sequences of VP1 are shown in Fig 2. We also investigated the amino acid variation occurring in the VP1 of these strains, especially in the predicted GII.4 blockade epitope sites A–E (Tables 2 and 3).

**Table 2:** Amino acid variation in blockade epitope of representative strains detected in GII.4 outbreaks in Yokohama, Japan.

				Epitope A						Epitope B		Epitope C		Epitope D			Epitope E		
Variant type	Strain^a^	Collection	Place	294	296	297	298	368	372	333	382	340	376	393	394	395	407	412	413
Yerseke 2006a	Yerseke38	2006		A	T	Q	E	S	S	V	R	R	E	S	T	T	D	D	S
DenHaag89 2006b	DenHaag89	2006		A	S	R	N	S	E	V	K	G	E	S	T	T	S	N	V
	**y06-V82-2**	**Jan-2007**	**Nursing home**	**.**	**.**	**.**	**.**	**.**	**.**	**.**	**.**	**.**	**.**	**.**	**.**	**.**	**.**	**.**	**.**
	**y06-V110-5**	**Feb-2007**	**Nursing home**	**.**	**.**	**.**	**.**	**.**	**.**	**.**	**.**	**.**	**.**	**.**	**.**	**.**	**.**	**.**	**.**
	**y07-V179-1**	**Jan-2008**	**Nursing home**	**.**	**.**	**.**	**.**	**.**	**.**	**.**	**.**	**.**	**.**	**.**	**.**	**.**	**.**	**.**	**A**
	**y07-V203-1**	**Mar-2008**	**Nursing home**	**.**	**.**	**.**	**.**	**.**	**.**	**.**	**.**	**A**	**.**	**.**	**.**	**.**	**.**	**.**	**.**
	**y08-207-2**	**Nov-2008**	**Banquet hall**	**.**	**.**	**.**	**.**	**.**	**.**	**.**	**.**	**.**	**.**	**G**	**.**	**.**	**.**	**.**	**.**
	**y08-V290-2**	**Dec-2008**	**Nursing home**	**.**	**.**	**.**	**.**	**.**	**.**	**.**	**.**	**.**	**.**	**.**	**.**	**.**	**.**	**.**	**.**
	**y08-238-2**	**Jan-2009**	**Banquet hall**	**.**	**.**	**.**	**D**	**.**	**.**	**.**	**.**	**.**	**.**	**.**	**.**	**.**	**.**	**D**	**.**
	**y09-188-98**	**Dec-2009**	**Banquet hall**	**.**	**.**	**.**	**.**	**G**	**.**	**.**	**.**	**.**	**.**	**.**	**.**	**.**	**.**	**.**	**.**
	**y09-205-1**	**Jan-2010**	**Restaurant**	**.**	**.**	**.**	**.**	**.**	**.**	**.**	**.**	**.**	**.**	**G**	**.**	**.**	**.**	**D**	**.**
	**y10-V475-1**	**Dec-2010**	**Nursery**	**.**	**.**	**.**	**.**	**.**	**.**	**.**	**.**	**.**	**.**	**G**	**.**	**.**	**.**	**.**	**.**
	**y10-V526-8**	**Jan-2011**	**Nursing home**	**.**	**.**	**.**	**.**	**.**	**.**	**M**	**.**	**.**	**.**	**.**	**.**	**.**	**.**	**.**	**.**
	**y11-188-1**	**Dec-2011**	**Banquet hall**	**.**	**.**	**.**	**.**	**G**	**.**	**.**	**.**	**.**	**.**	**G**	**.**	**.**	**.**	**.**	**.**
	**y11-V664-1**	**Jan-2012**	**Nursing home**	**.**	**.**	**G**	**.**	**.**	**.**	**M**	**.**	**.**	**.**	**N**	**.**	**.**	**.**	**.**	**.**
	**y11-V689-2**	**Feb-2012**	**Nursing home**	**.**	**.**	**.**	**.**	**.**	**.**	**.**	**.**	**.**	**.**	**.**	**.**	**.**	**.**	**.**	**.**
	Taoyuan/CGMH55	Feb-2012		.	.	.	.	E	.	M	.	.	.	G	.	.	.	.	.
	**y12-V758-3**	**Nov-2012**	**Nursery**	**.**	**.**	**.**	**.**	**E**	**.**	**M**	**.**	**.**	**.**	**G**	**.**	**.**	**.**	**.**	**.**
	**y12-V836-3**	**Dec-2012**	**Nursing home**	**.**	**.**	**.**	**.**	**E**	**.**	**M**	**.**	**.**	**.**	**G**	**.**	**.**	**.**	**.**	**.**
Apeldoorn 2007	Apeldoorn317	Dec-2007		T	S	R	N	A	D	V	K	T	D	D	T	A	S	N	N
New Orleans 2009	New Orleans1805	Nov-2009		P	S	R	N	A	D	V	K	T	E	S	T	T	S	N	I
Sydney 2012	NSW0514	Mar-2012		T	S	R	N	E	D	V	K	T	E	G	T	T	S	N	T
	AlbertaEI337	Sep-2011		.	.	.	.	.	.	.	.	A	.	S	.	.	.	.	.
	PA363	Nov-2011		.	.	.	.	.	.	M	.	.	.	S	.	.	.	.	.
	**y11-V615-5**	**Nov-2011**	**Nursery**	**.**	**.**	**.**	**.**	**.**	**.**	**.**	**.**	**.**	**.**	**.**	**.**	**.**	**.**	**.**	**.**
	**y11-V627-3**	**Dec-2011**	**Nursery**	**.**	**.**	**.**	**.**	**.**	**.**	**.**	**.**	**.**	**.**	**.**	**.**	**.**	**.**	**.**	**.**
	**y11-V633-3**	**Dec-2011**	**Primary school**	**.**	**.**	**.**	**.**	**.**	**.**	**.**	**.**	**.**	**.**	**.**	**.**	**.**	**.**	**.**	**.**
	**y11-V645-27** ^b^	**Dec-2011**	**Restaurant**	**.**	**.**	**.**	**.**	**.**	**.**	**.**	**.**	**.**	**.**	**.**	**.**	**.**	**.**	**.**	**.**
	**y11-V648-1**	**Dec-2011**	**Nursing home**	**.**	**.**	**.**	**.**	**.**	**.**	**.**	**.**	**.**	**.**	**.**	**.**	**.**	**.**	**.**	**.**
	**y12-V753-1**	**Oct-2012**	**Nursing home**	**.**	**.**	**.**	**.**	**.**	**.**	**.**	**.**	**.**	**.**	**S**	**.**	**.**	**.**	**.**	**.**
	**y12-V762-1**	**Nov-2012**	**Nursery**	**.**	**.**	**.**	**.**	**.**	**.**	**.**	**.**	**.**	**.**	**S**	**.**	**.**	**.**	**.**	**.**
	**y12-V780-1**	**Nov-2012**	**Primary school**	**.**	**.**	**H**	**.**	**.**	**N**	**.**	**.**	**.**	**.**	**.**	**.**	**.**	**.**	**.**	**.**
	**y12-V812-2**	**Dec-2012**	**Nursing home**	**.**	**.**	**.**	**.**	**.**	**.**	**.**	**.**	**.**	**.**	**.**	**.**	**.**	**.**	**.**	**.**
	**y12-V854-3**	**Dec-2012**	**Hospital**	**.**	**.**	**.**	**.**	**.**	**.**	**.**	**.**	**.**	**.**	**.**	**.**	**.**	**.**	**.**	**.**
	**y12-V849-2**	**Jan-2013**	**Nursing home**	**.**	**.**	**.**	**.**	**.**	**.**	**.**	**.**	**.**	**.**	**.**	**.**	**.**	**.**	**.**	**.**
	**y13-140-1**	**Nov-2013**	**Banquet hall**	**.**	**.**	**.**	**.**	**.**	**.**	**.**	**.**	**.**	**.**	**.**	**.**	**.**	**.**	**.**	**.**
	**y13-V938-3**	**Nov-2013**	**Nursery**	**.**	**.**	**.**	**.**	**.**	**.**	**.**	**.**	**.**	**.**	**.**	**.**	**.**	**.**	**.**	**.**
	**y13-166-1**	**Dec-2013**	**Catering**	**.**	**.**	**.**	**.**	**.**	**.**	**.**	**.**	**.**	**.**	**.**	**.**	**.**	**.**	**.**	**.**
	**y13-V988-5**	**Dec-2013**	**Nursing home**	**.**	**.**	**.**	**.**	**.**	**.**	**.**	**.**	**.**	**.**	**S**	**.**	**.**	**.**	**.**	**.**
	**y13-V1010-1**	**Jan-2014**	**Nursing home**	**.**	**.**	**.**	**.**	**.**	**.**	**.**	**.**	**.**	**.**	**.**	**.**	**.**	**.**	**.**	**.**

^a^ The strains shown in bold font were analyzed in this study.

^b^ This strain was detected in a mixed GI and GII outbreak.

**Table 3:** Number of amino acid variable sites in VP1 compared with prototype strains.

				No. (%) of variable sites^a^	
Domain	Subdomain	AA positions	No. of amino acids	DenHaag 2006b strains (n = 16) during 7 seasons	Syendy 2012 strains (n = 16) during 3 seasons
Shell		41–222	182	3 (1.6)	0 (0)
Protrusion	P1	223–273, 402–531	181	7 (3.9)	3 (1.7)
	P2	274–401	128	13 (10.2)	7 (5.5)
Total			541	25 (4.6)	14 (2.6)

^a^ Variable sites were defined by the presence of an amino acid substitution in the alignment of at least one strain.

### Analysis of 2006b strains

Sixteen of the 2006b strains that were detected in Yokohama were analyzed. They showed 96.2–99.2% nucleotide sequence identity and 98.7–99.8% deduced amino acid sequence identity with the 2006b prototype, DenHaag89 strain (EF126965). A phylogenetic tree constructed using the amino acid sequence of the whole VP1 gene showed that the 2006b strains, including our strains, formed a separate cluster from the Yerseke 2006a, Apeldoorn 2007, New Orleans 2009, and Sydney 2012 variants (Fig 2). When the amino acid variation of VP1 in the alignment of these 16 strains was investigated, 25 sites (4.6% of the 541 amino acids) were substituted during seven seasons (from the 2006–2007 season to the 2012–2013 season). Of these, 13 sites were located in the P2 subdomain, and the P2 subdomain had a higher percentage of substitutions than other domains (Table 3).

Out of the 16 detected 2006b strains, 12 strains had some amino acid substitutions in the A–E epitopes compared with DenHaag89 (Table 2). These substitutions were largely concentrated in three residues (amino acid (aa)368 in epitope A, aa333 in epitope B, and aa393 in epitope D). Both y12-V758-3 and y12-V836-3, which were identified from the two 2006b outbreaks in the 2012–2013 season, had three amino acid substitutions, from S to E at aa368 (S368E) of epitope A, V333M of epitope B, and S393G of epitope D, compared with DenHaag89. Notably, this substitution in epitope A was not observed in the other strains analyzed in this study from the seasons between 2006 and 2012, and these substituted amino acids of epitope A and epitope D were consistent with the residues at the same site of the Sydney 2012 prototype, NSW0514 strain (JX459908). Moreover, y12-V836-3 showed the highest nucleotide sequence identity (98.7%) in VP1 to the Taiwanese strain Taoyuan/CGMH55 (KC517366), which was detected in February 2012 by a BLAST search. This Taiwanese strain had the same amino acids as y12-V758-3 and y12-V836-3 in epitopes A–E (Table 2).

### Analysis of Sydney 2012 strains

Sixteen of the Sydney 2012 strains that were detected in Yokohama were analyzed. They showed 98.6–99.1% nucleotide sequence identity and 98.9–99.6% amino acid sequence identity with the Sydney 2012 prototype, NSW0514. A phylogenetic tree constructed using the amino acid sequences showed that all five strains detected in Yokohama in the 2011–2012 season clustered away from the other Sydney 2012 strains detected in Yokohama in the 2012–2013 and 2013–2014 seasons (Fig 2). Fourteen sites in VP1 (2.6% of the 541 amino acids) were substituted during three seasons (from the 2011–2012 season to the 2013–2014 season). Of these, seven sites (5.5% of the amino acids in the P2 subdomain) were located in the P2 subdomain. In contrast, no variable sites were detected in the shell domain (Table 3).

Compared with the prototype Sydney 2012 strain NSW0514, none of the sixteen detected Sydney 2012 strains had any substitutions in epitopes B, C, or E (Table 2). It has also been reported that early Sydney 2012 strains, Canadian strain AlbertaEI337 (KF509947) and Italian strain PA363 (KF668567), were detected in the 2011–2012 season [5, 25, 26]. Compared with NSW0514, AlbertaEI337 had differences in epitope C and epitope D and PA363 had differences in epitope B and epitope D. However, we did not observe any differences from NSW0514 in the blockade epitopes of the five strains that were detected in the 2011–2012 season (Table 2).

## Discussion

NoVs are the most common cause of gastrointestinal disease outbreaks [1]. They can spread through contaminated food or water or from person to person and are highly infectious [1, 2]. Consequently, NoV is a public health problem worldwide, including in Japan. In this study, to better understand the trends of NoV strains circulating in the population of Yokohama, Japan, the strains associated with outbreaks in various settings as well as the epidemic variation in NoV GII.4 strains were investigated in the seasons between 2006 and 2014.

GII was the predominant genogroup throughout the eight seasons in our study and was detected in 88.2% of the NoV-positive outbreaks. The proportion of GII was consistent with those found in outbreak studies performed in the United States of America and in Canada [5, 17], whereas it was lower than those shown in sporadic studies [27, 28].

For GII genotypes, although GII.4 was the most predominant among the 22 different GII genotypes in all except the 2010–2011 season, the GII.4 proportion of GII differed by season. When the prevalence of GII genotypes other than GII.4 is considered, the endemic genotypes of the 2008–2009, 2009–2010, 2010–2011, 2011–2012, and 2013–2014 seasons were GII.6, GII.2, GII.2 and GII.3, GII.14, and GII.6, respectively. These four genotypes are mainly associated with sporadic infections in children in some countries, including Japan during this study period [7, 27, 29–31]. Increasing numbers of gastroenteritis samples have been tested in the past years [32]. Additionally, the number of reported outbreaks in primary schools and childcare facilities has also increased during this period [32], resulting in an increase in the detection of GII.6, GII.2, GII.3, and GII.14 strains. Additionally, in Yokohama during the 2008–2009 season, GII.6 was the second most dominant genotype after GII.4. This is consistent with the epidemic status of Shizuoka, Japan, in which the emergence of a new norovirus GII.6 variant was reported in 2008–2009 [33]. A phylogenetic analysis using region C [data not shown] showed that this variant strain and most of the GII.6 strains detected in the 2008–2009 season in Yokohama belong to same subcluster.

The 2006–2007 season was the first season when the GII.4 variant 2006b was predominant. Similarly, the 2012–2013 season was the first season when 2012 Sydney was predominant. The increasing numbers of GII.4 outbreaks in the 2006–2007 and 2012–2013 season were likely related to the emergence of these two variants, and they spread rapidly because the population did not have herd immunity for these two variants. Similar to findings in the rest of the world, the proportion of GII.4 was relatively high in the 2006–2007 and 2012–2013 seasons in Yokohama [4, 5, 27]. In contrast, several studies have reported that New Orleans 2009 was predominant in some seasons [5, 6, 17]. However, this variant was not predominant in Yokohama during any of the seasons in this study, although New Orleans 2009 was detected during three seasons since December 2009. This finding is consistent with the studies of other Asian countries [27, 31]. The prevalence of New Orleans 2009 might have been affected by the prevalence of 2003 Asia, another GII.4 variant. 2003 Asia, of which Sakai/04-179 (AB220922) is a prototype strain, was circulating from 2004 to 2005, mostly in Asia [4, 24, 28]. The duration of immunity to NoV has been estimated at 4.1 to 8.7 years in various models [34]. It is possible that the Asian population who had been infected with 2003 Asia NoV had antibodies with cross-reactivity with New Orleans 2009. However, this hypothesis cannot be confirmed at present because there have not been any studies published on antibodies to 2003 Asia variants. The Yerseke 2006a and Apeldoorn 2007 variants were rarely detected in this study, and this result agrees with the sporadic cases found in Asian and Russian studies that were conducted during the same period [7, 27, 35].

To date, 2006b is one of the pandemic variants, and currently Sydney 2012 is still a pandemic variant around the world [5–7, 17, 27]. In Yokohama, either 2006b or Sydney 2012 was the predominant variant of each season in this study period. Because of their predominance, we used representative strains of 2006b and Sydney 2012 to investigate their genetic characteristics. A phylogenetic tree constructed using the amino acid sequence coding VP1 revealed that the 2006b strains in Yokohama are distantly related to other GII.4 variants. The 2006b strains had an accumulation of mutations in the capsid P2 subdomain compared with the variants that emerged before 2006b [24, 36]. Antigenic variation is an important factor contributing to NoV GII.4 evolution [13]. This huge shift of amino acids in VP1, especially in the P2 subdomain, is believed to be one of the factors that allowed the 2006b variant to escape existing herd immunity. Additionally, it has been reported that, in Brazil, the 2006b variant strains were grouped into two remarkably separate sub-clusters between the samples collected during 2006–2008 and those collected during 2009–2011 [18]. However, our 2006b strains, which were collected from the 2006–2007 to the 2012–2013 seasons did not separate into any obvious sub-clusters. The number of amino acid substitutions in the blockade epitope of our 2006b strains compared with the prototype trended upward over the course of this study. Notably, two 2006b strains detected in the 2012–2013 season had glutamic acid at aa368 of epitope A and glycine at aa393 of epitope D, which are consistent with the residues at the same site of the Sydney 2012 prototype. It is thought that epitope A contributes to antigenic differences and epitope D affects Histo-Blood Group Antigens (HBGAs) binding [14]. Epitopes A and D may be the major drivers of escape from herd immunity in contemporary strains, and screening new strains for changes in these epitopes may provide a quick and valuable method for effective vaccine design and reformulation [37].

In Yokohama, the Sydney 2012 variant replaced the 2006b variant in the 2012–2013 season and expanded rapidly, although the first appearance of the Sydney 2012 variant was in the 2011–2012 season. Similarly, it has also reported that the Sydney 2012 variant became predominant in the next winter season after its first appearance in Italy and Canada [5, 25, 26]. These Italian and Canadian early Sydney 2012 strains had some mutations in the blockade epitopes compared with NSW0514, and these strains likely played a role in the evolution / adaptation of the novel pandemic variant before spreading worldwide [5, 25]. However our five early strains did not have these mutations in the blockade epitopes. Furthermore, our Sydney 2012 strains had little the antigenic diversity, although all five strains detected in the 2011–2012 season were phylogenetically separate from the other Sydney 2012 strains detected in the 2012–2013 and 2013–2014 seasons. However, if the prevalence of Sydney 2012 continues on a path similar to that of 2006b, the Sydney 2012 strains will probably accumulate additional substitutions. We need continuous surveillance of Sydney 2012 to determine the magnitude and mechanism of the pandemic caused by this variant because this variant still causes epidemics.

In conclusion, we identified GII.4 variants from outbreaks in various settings that occurred in Yokohama, Japan, over eight NoV seasons. The prevalence of some of the GII.4 variant types differs between Asia and other parts of the world, and we found amino acid substitutions among GII.4 variants. We believe that, although our work is limited geographically to Yokohama, Japan, these data will lead to a better understanding of the NoV requirement for constant changes in their host-binding site to escape the immune response. To our knowledge, this is the first investigation to conduct long-term monitoring of GII.4 variants in Yokohama, Japan. Many points about norovirus epidemics and evolution remain unknown. To elucidate these, steady surveillance and an accumulation of data are needed for future analyses.
